# XFEM for Composites, Biological, and Bioinspired Materials: A Review

**DOI:** 10.3390/ma17030745

**Published:** 2024-02-04

**Authors:** Andre E. Vellwock, Flavia Libonati

**Affiliations:** 1 B CUBE—Center for Molecular Bioengineering, Technische Universität Dresden, 01307 Dresden, Germany; andre.eccel_vellwock@tu-dresden.de; 2 Department of Mechanical, Energy, Management and Transportation Engineering, University of Genoa, 16145 Genoa, Italy

**Keywords:** XFEM, FEM, fracture modeling, composites, biological materials, bioinspiration, AI

## Abstract

The eXtended finite element method (XFEM) is a powerful tool for structural mechanics, assisting engineers and designers in understanding how a material architecture responds to stresses and consequently assisting the creation of mechanically improved structures. The XFEM method has unraveled the extraordinary relationships between material topology and fracture behavior in biological and engineered materials, enhancing peculiar fracture toughening mechanisms, such as crack deflection and arrest. Despite its extensive use, a detailed revision of case studies involving XFEM with a focus on the applications rather than the method of numerical modeling is in great need. In this review, XFEM is introduced and briefly compared to other computational fracture models such as the contour integral method, virtual crack closing technique, cohesive zone model, and phase-field model, highlighting the pros and cons of the methods (e.g., numerical convergence, commercial software implementation, pre-set of crack parameters, and calculation speed). The use of XFEM in material design is demonstrated and discussed, focusing on presenting the current research on composites and biological and bioinspired materials, but also briefly introducing its application to other fields. This review concludes with a discussion of the XFEM drawbacks and provides an overview of the future perspectives of this method in applied material science research, such as the merging of XFEM and artificial intelligence techniques.

## 1. Introduction

The finite element method (FEM) has revolutionized material design by reducing redesign time and costs through prefabrication and structural optimization. The FEM facilitates the assessment of mechanical properties, such as the maximum load a bridge can withstand and where the failure point might be, and the number of cycles an automobile suspension can last before discontinuity. Understanding the fracture behavior (e.g., crack nucleation and propagation) is essential. Yet, the standard FEM cannot fully model fracture. In the last few decades, a few methods have emerged to cover this gap. Perhaps the first, after observation of stress around cracks [1], the contour integral method (CIM) and its variations have been proposed to calculate the stress intensity factor on cracked media [2,3]. Embedded on commercial software, and directly related to CIM, J-integral [4], C_T_-integral [5], and T-stress [6] are available. Despite clear advantages such as robustness, calculation speed, and simplicity, the CIM requires the modeling of the crack plane or line, specification of its front [7], and careful mesh preparation. Moreover, it gives only a step-wise output as it does not simulate the propagation path. The virtual crack closing technique (VCCT), developed in 1977 [8], calculates the energy release rate and propagation mode of a pre-defined crack. Besides, it can simulate the crack path on commercial software, an improvement from the CIM. However, the VCCT is a numerically challenging method and can only simulate brittle fracturing [9]. Thus, it is commonly employed on simpler models such as for delamination and debonding case studies. The cohesive zone element (CZM) [10,11,12] applies cohesive regions to understand non-linear behavior on the front of a pre-defined crack. The CZM also requires that the crack propagation path be pre-set. Despite moderate numerical convergence [13], these requirements limit its applicability to crack modeling. In this sense, the CZM, like the VCCT, is mainly utilized for debonding analyses [14,15,16]. The phase-field model (PFM) was introduced in 2008 [17], quickly followed by another publication in 2010 [18]. The PFM is surpassing the CIM, VCCT, and CZM because it employs a scalar field (i.e., the phase field) to smoothly transition from the intact to the damaged element and does not consider the crack as a physical discontinuity [19,20]. In addition, the PFM can model multiple cracks and their branching and merging [21]. However, issues such as diffuse damage profile due to regularization length are often encountered [22,23], requiring proper user input to overcome it [23]. Currently, the PFM’s direct implementation in commercial software is absent and requires user input subroutines [20,24,25], reducing its popularization among the fracture mechanics community. A summary of the pros and cons of numerical methods that can simulate fracture behavior is available in Table 1.

The eXtended finite element method (XFEM) has been pushing the boundaries of fracture numerical analysis since its creation in 1999 [26]. The method is a powerful tool to simulate the fracture behavior of materials, elucidating the crack initiation and propagation under particular conditions. Frequently, the XFEM is applied to evaluate the influence of a material microstructure (e.g., different material properties and complex inner geometries) on the crack path, demonstrating hidden fracture toughness mechanisms and facilitating the design of mechanically superior materials. Naturally, composites are a direct application, where the XFEM can be used to alter the composite components towards a better material. In addition, biological and bioinspired materials have also been in the spotlight. Analyzing the fracture mode with the XFEM in biological materials, such as cortical bone and teeth, has not only allowed a better understating of the natural material mechanical characteristics but also the development of extraordinary bioinspired designs. These new architectures have been expanding the limits of structural materials by combining the usage of components with contrasting mechanical properties and creating complex innovative material designs. The novelty of this review is the comprehensive synopsis of the latest application of the XFEM in the field of composites and biological and bioinspired materials. The limitations of the method, such as convergence issues, are outlined. In the following sections, short- to medium-term outlooks are discussed, comprising 3D XFEM simulations, possible new applications, the usage of components with non-linear material properties, and the XFEM and artificial intelligence.

## 2. The eXtended Finite Element Method

The method was first described by Belytschko and Black in 1999 [26] and improved by Moës, Dolbow, and Belytschko in the same year [30]. The authors aimed to develop a finite element method that could model crack formation and growth without the need to remesh the crack tip, thus drastically reducing the required computational power and other problems associated with meshing. In 1996, the partition of unity finite element method was introduced [31], where local enrichment functions are used to explain the crack tip singularity. The XFEM, based on this method, describes the displacement vector u as follows:

(1) $$u=\sum_{I=1}^{N}N_{I}\left(x\right)[u_{I}+H(x)\cdot a_{I}+\sum_{\alpha =1}^{4}F_{\alpha }(x)\cdot b_{I}]$$

**Figure 1:**
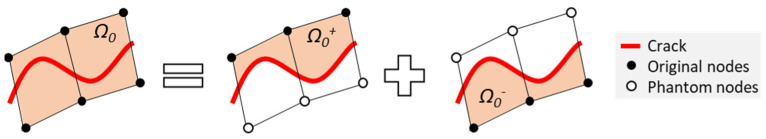
Superposition method. Phantom nodes are created to model the crack propagation, allowing for mesh-independent fracture modeling.

This formula shows that $N_{I}\left(x\right)$, the standard finite element nodal shape functions, are enriched by the jump function, H(x), and the asymptotic crack tip function, $F_{\alpha }(x)$. The function H(x) computes the distance from a sample point, $\left(x\right)$, to the nearest crack point, $\left(x^{*}\right)$, multiplied by the unit vector normal to the crack tip at $x^{*}$. If $(x-x^{*})\cdot n\geq 0$, $H\left(x\right)=1$, otherwise $H\left(x\right)=-1$. The crack tip function is defined using polar coordinates regarding the crack tip:

(2) $$F_{\alpha }\left(x\right)=[\sqrt{r}\cdot sin\frac{\theta }{2},\;\sqrt{r}\cdot cos\frac{\theta }{2},\;\sqrt{r}\cdot sin\theta \cdot sin\frac{\theta }{2}\;,\;\sqrt{r}\cdot sin\theta \cdot cos\frac{\theta }{2}]$$

where *θ* is set as zero when tangential to the tip. To facilitate the computation of moving cracks, current simulation software applies the concept of phantom nodes (Figure 1) [27]. In this technique, a mesh element that is in the path of the crack is replaced by two twin elements, where the original nodes are interchanged into phantom nodes. The original physical domain, Ω, is also divided into two, as ${\Omega_{0}}^{+}$ and ${\Omega_{0}}^{-}$, where superposition occurs: $\Omega ={\Omega_{0}}^{+}+{\Omega_{0}}^{-}$. Given a previously sufficiently refined mesh, phantom nodes allow for mesh-independent crack modeling [27,28,29].

### 2.1. Crack Initiation

In commercial software, the location of crack initiation can be either preset, by modeling the crack with a user-established length, or computer-calculated based on one or more set criteria (e.g., maximum principal stress criterion, maximum principal strain criterion, quadratic principal stress criterion, quadratic nominal strain criterion, etc.). After the crack propagation, the same factors are used to compute the direction of growth. For example, if the maximum principal stress criterion is selected, a crack will initiate or grow orthogonally to the location where the criterion is achieved when $f=\left\{\langle \sigma_{max}\rangle |{\sigma_{max}}^{0}\right\}\geq 1$, where $\langle \sigma_{max}\rangle$ is the maximum non-compressive principal stress and ${\sigma_{max}}^{0}$ is the maximum allowable principal stress.

### 2.2. Crack Propagation

The fracture propagation is based on a material traction–separation model, where the normal traction stress vector, t, is written based on its normal, $t_{n}$, and two shear parts, $t_{s}$ and $t_{t}$, and is related to the material stiffness matrix, K, and the local separation, δ, according to the following equation:

(3) $$t=\left\{\begin{array}{c} t_{n}\\ t_{s}\\ t_{t} \end{array}\right\}=\left[\begin{array}{ccc} K_{nn} & 0 & 0\\ 0 & K_{ss} & 0\\ 0 & 0 & K_{tt} \end{array}\right]\cdot \left\{\begin{array}{c} \delta_{n}\\ \delta_{s}\\ \delta_{t} \end{array}\right\}=K\cdot \delta$$

After the crack initiation criteria are achieved, the material damage evolution is governed by a damage variable, D, where D=0 if the crack has not started to evolve and D=1 if the crack has fully evolved. This variable is related to the normal traction stress vector components as follows:

(4) $$t_{n}=\left\{\begin{array}{c} \left(1-D\right)\cdot T_{n},\;\;if\;T_{n}\geq 0\\ T_{n},\;\;\;if\;T_{n}<0 \end{array}\right.$$

(5) $$t_{s}=\left(1-D\right)\cdot T_{s}$$

(6) $$t_{t}=\left(1-D\right)\cdot T_{t}$$

where T is the predicted stress, taking into consideration the traction–separation elastic behavior and present separations disregarding the damage.

### 2.3. Availability

The method is not only available in commercial software such as Abaqus since version 6.9 (Simulia, Providence, RI, USA) and Ansys 17.2 (Canonsburg, PA, USA) but also in free software such as GetFEM 5.4.2 [32] and Code_Aster 14.6 [33]. Modifications of the XFEM have also been proposed [34,35,36,37,38,39].

## 3. XFEM for Material Design

### 3.1. XFEM and Composites

**Figure 2:**
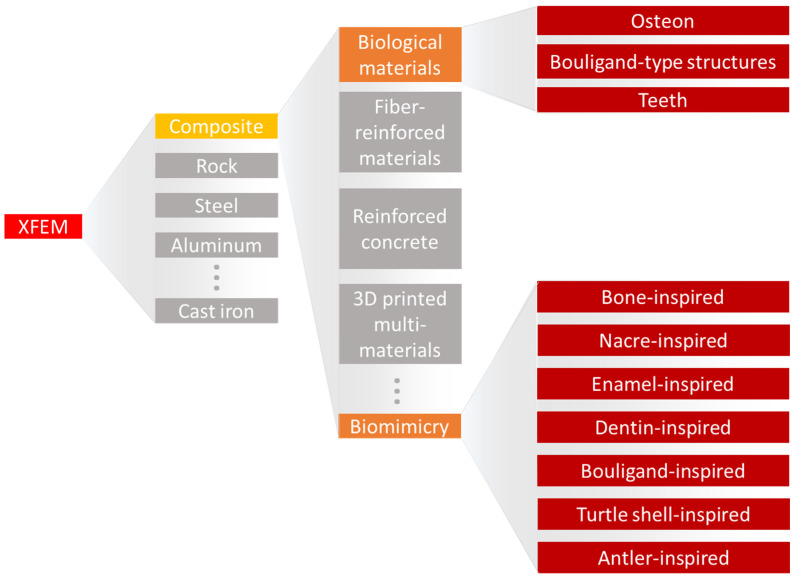
A diagram of XFEM usage in material simulations, highlighting its application to the field of biomaterials and bioinspired materials.

The XFEM has been applied to simulate fracture behavior in a variety of materials (Figure 2), ranging from rocks [40,41,42,43] to steel [44,45,46,47,48], cast iron [49,50], aluminum [51,52], silicon oxycarbide [53,54], and composites [55,56,57]. In the steel industry, the XFEM has been applied to better understand fatigue crack propagation on bridges [44], the impact properties of high-pressure gas transportation pipes [45], and the flexural response of coated steel used for yacht manufacturing [48]. Considering cast iron, Chang et al. [49] showed that the XFEM results were in great agreement with experimental uniaxial tests, demonstrating the material elastoplastic behavior. Applying a complex loading condition, with an eccentric impact load on a three-point bending cast iron structure, Tsuda et al. [50] highlighted the flexibility of the XFEM in uncommon cases. For an aluminum alloy sample, Gairola and Jayaganthan [51] correctly predicted the material fracture toughness, suggesting that 3D XFEM simulations are slightly better than 2D. Kim et al. [58] showed the effects of hydrogen gas on the fracture behavior of an aluminum alloy by simulating fracture resistance curves of compact tension specimens. The XFEM load and displacement curves were within 5% of the experimental conditions. Recently, Xie et al. [53] captured the mechanical and fracture behavior of a silicon carbide film, where the XFEM was a successful method for evaluating interfacial strength.

Composites is a growing field that is based on the design of materials with two or more components to create multi-material structures with particular mechanical properties. For example, the construction industry uses reinforced concrete as a load-bearing structure in the development of buildings [59]. Reinforced concrete refers to the use of aligned metallic bars in a concrete mix, where the bars play the essential role of increasing the composite’s overall load-bearing capacity. The modeling of columns made of reinforced concrete showed that the XFEM can predict the start location and growth pattern of cracks when the structure is subjected to monotonic or cyclic loadings [60,61]. Chung et al. [62] simulated a bending test of a reinforced concrete beam and demonstrated that the XFEM was able to initiate cracks in the majority of the locations experimentally observed. Moreover, the authors discussed problematics about the method’s convergence which were possibly accentuated by the choice of a 3D model over a simplified 2D. Marzec and Bobiński [63] studied the influence of cohesive softening law on XFEM simulations of concrete beams under bending. The authors applied three softening cases: bilinear, exponential, and a rational Bezier, concluding that all produced consistent results if different fracture energies are considered.

In the composite field, the method has been extensively used to study fiber-reinforced materials, such as in studies that evaluated the influence of random short fiber inclusions in composites, considering 2D [64] and 3D simulations [65]. Pike and Oskay [64] implemented a modified framework to understand the effect of random fiber inclusions, comparing the method to standard finite element modeling in case studies such as single fiber inclusion and random short fiber composites. In another study, Pike and Oskay [65] successfully showed that the influence of short fibers on composite materials can be modeled in a 3D simulation, rather than the previous 2D one. Serna Moreno et al. [66] showed that in bi-axial tensile tests of chopped glass-reinforced composites, the XFEM simulated crack start location, path, and even growth velocity in agreement with the experimental results. XFEM fatigue simulation on similar bi-axial samples demonstrated the capacity of the method to analyze fracture behavior under complex loads [67]. Fracturing is a large issue for composite structures. In thin plates where buckling can easily occur, the issue is also present. Simulations of cracks in composite plates using the XFEM facilitated the analysis of the crack lengths and angles, fiber directions, and even boundary conditions on overall structural behavior during buckling [68].

Delamination is one of the most crucial damages that can occur on impacted composites [69]. It refers to a de-adhesion of adjacent components, such as the peeling of a coating on a substrate and the detachment of fibers in a matrix. Despite a local effect, it can compromise the capacity of a whole structure to withstand loading. Carbon fiber composites and glass fiber-reinforced aluminum laminates are widely applied to the aerospace and high-end automotive industries. In this sense, Abdullah et al. [70] demonstrated that the XFEM is a useful tool to simulate delamination in carbon fiber composites with different fiber orientations, displaying the orientation influence on the crack path. Moreover, the XFEM fracture behavior agreed with the experimental results. Curiel Sosa and Karapurath [71] obtained similar results while simulating delamination in fiber metal laminates. The authors also suggested the XFEM as a computational time-saving tool and reinforced that the method is independent of the finite element size.

### 3.2. XFEM and Porous Materials

**Figure 3:**
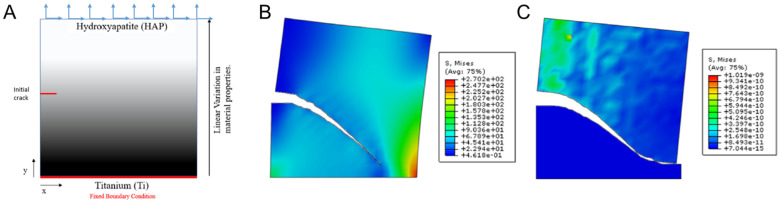
The influence of porosity on a crack path. (**A**) Assigned material properties and boundary conditions to the pre-cracked plate, highlighting the varying properties from titanium to hydroxyapatite. Crack propagation behavior for (**B**) non-porous and (**C**) porous conditions in the functionally graded structure. Reproduced with permission from [76]; copyright Elsevier.

Porous materials are widely observed in natural materials and engineering due to their desirable properties that emerge from their porosities, such as large specific surface area [72,73], energy absorption [74], and heat insulation [75]. Porosity and bulk material properties directly affect fracture behavior and the overall behavior of porous materials. Singh et al. [76] modeled a functionally graded plate with a linear variation in material and mechanical properties and studied the failure mode under shear loading (Figure 3A). The plate-assigned materials varied from hydroxyapatite to titanium, a condition often seen in fabricated bioimplants. The results highlighted that for both the non-porous (Figure 3B) and the porous (Figure 3C) conditions, the crack path tended toward the titanium region. However, in the porous plate, the crack path took a slightly longer path. He and Zhuang [77] applied the XFEM to study hydraulic crack propagation in porous media. Hydraulic fracturing, or fracking, is a process used to extract underground gas and oil for the energy industry. In this study, the XFEM was successfully applied to analyze fluid–structure interaction by modeling crack propagation under pressurized zones in porous media. Rezanezhad et al. [78] also evaluated the XFEM on porous rocks, displaying the influence of pore size, orientation, and distance on crack propagation. Salimzadeh and Khalili [40] simulated fracking by a coupled hydro–poroelastic model, incorporating the injected fracturing fluid and the host fluids. The XFEM and the cohesive elements were able to model the fracture discontinuity and propagation path.

### 3.3. XFEM and Biological Materials

**Figure 4:**
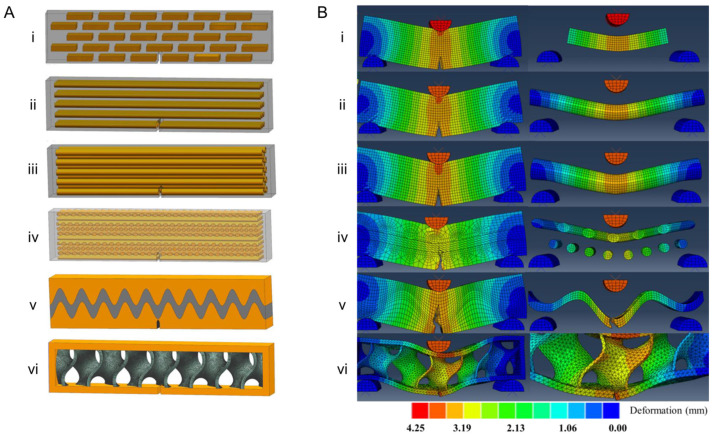
Mechanical behavior of composite designs inspired by six types of commonly found natural topologies: (**i**) Layered, (**ii**) Columnar, (**iii**) Tubular, (**iv**) Helical, (**v**) Sutured, and (**vi**) Sandwich. (**A**) Biological material designs and (**B**) their corresponding fracture behavior under bending conditions. Reproduced from [93] under Creative Commons CC BY.

Nature-made materials have incredible properties such as color shifting [79], self-cleaning [80,81], tissue regeneration [82], and the capacity to resist fracture [83,84]. Significant findings in the fracture behavior of biological materials have also been recently in the spotlight. For instance, the mantis shrimp strikes its prey by releasing an extreme amount of energy from its saddle [85]. Its club-like appendage, known as the dactyl club, is responsible for direct contact with the prey. It is heavily mineralized with specific microstructural architecture to dissipate energy, reducing possible self-inflicted damage [86,87]. Spiders can attach to a variety of surfaces due to their dual attachment system. For smooth surfaces, small hairs located on the tip of their tarsus facilitate organism adhesion through van der Waals forces. Nevertheless, for rougher surfaces, these adhesion forces are not strong enough, and the animal uses its claws to increase the friction with the surface. The claw is made of a hierarchical material with metal ion inclusions, creating a high wear-resistance structure [88]. Mammal bones, in particular, the cortical bone tissue, are load-bearing structures that consist—at the microscale—mostly of osteons, a cylindrical structure with a concentric inner channel surrounded by weaker outer layers called cement lines. Due to its architecture, fracture toughness mechanisms such as crack deflection and arrest occur, increasing considerably the overall fracture toughness of the bone tissue [89,90,91,92]. The influence of the microstructure found in biological tissues on the properties of the macroscale material is evident. However, understanding the mechanisms behind the enhancement in mechanical properties is not trivial, and a combination of experimental and computational analysis is often needed. In this direction, a broad XFEM study evaluated six contrasting biological structures against bending loads (Figure 4) [93]. The selected designs are commonly found on nature-made materials: layered (e.g., insect exoskeletons and nacre shells), columnar (e.g., spider silk and tendons), tubular (e.g., dentin and horns), helical (e.g., mantis shrimp dactyl club), sutured (e.g., turtle shells), and sandwich (e.g., toucan beaks). Despite considering simplified designs made by only two components, i.e., soft and stiff building blocks, and other needed simplifications, this study was able to well predict every design fracture behavior. Moreover, it demonstrated the influence of the arrangement of soft–stiff components in biological materials on the mechanical properties. For example, the authors showed that the helical arrangement of columns deflects cracks in the material’s soft matrix structure (Figure 4A, condition iv). This study not only highlighted the XFEM’s performance on composites with complex designs but also provided inspiration to the design of bioinspired materials.

**Figure 5:**
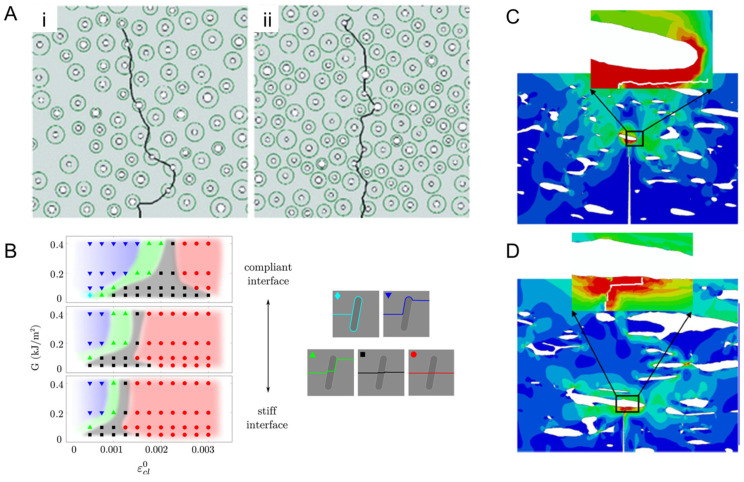
XFEM applied to the fracture analysis of cortical bone. (**A**) Crack propagation path on the cortical tissue with cement lines with altered elastic moduli: (**i**) 25% lower and (**ii**) same as that of an osteon. Reproduced with permission from [94]; copyright Springer Nature. (**B**) The observed crack configuration-related simulation parameters: strain energy release rate (G) and critical interface strain (ε) reproduced from [95] under Creative Commons CC BY. (**C**) Younger bone can deflect the crack, while (**D**) cannot. Reproduced with permission from [98]; copyright Elsevier.

The XFEM applied to the study of cortical bone has been essential to demonstrate that local material heterogeneity is the backbone of the crack propagation path [92,94,95,96]. Li et al. [94] showed that the cement line, i.e., the sheath around the osteon, contributes to deflecting and arrest more cracks when its elastic modulus is 25% lower than that of the osteon. This behavior directly increases the fracture toughness of the material (Figure 5A). Gustafsson et al. [95] not only reaffirmed this result but also demonstrated that decreasing the interface strength of the cement line also contributes to an increase in the frequency of crack deflection. A more compliant interface indeed favors the fracture path that goes around the cement line, as shown by the light and dark blue configurations in Figure 5B.

Modeling a composite inner structure is essential to simulate its fracture. However, most of the studies tend to simplify the bone microstructure to facilitate its computational analysis. For example, only one perfectly circular osteon is generally modeled. In a different path, Gustafsson et al. [96] applied X-ray microtomography to extract the bone local tissue orientation maps which were used as input to an XFEM analysis. The innovative method showed very realistic crack paths when compared to the experimental counterparts [97]. Similarly, Yadav et al. [98], based on microtomography data of the cortical bone, showed that crack behavior varies on the bone age. The authors applied nanoindentation measurements to extract the properties of the osteon, cement line, and interstitial matrix. Despite their results showing only small variations in the mechanical properties of young and old bone, the structural comparison highlighted significant points: aged bone has 15% less bone volume fraction and 80% smaller osteon wall thickness. When implemented into the XFEM model, the transversal crack propagates along the cement line in young bone (Figure 5C). Meanwhile, for old bone, the crack crosses the osteon (Figure 5D). This has also been observed in different anatomical bone regions and is correlated to the local loading and the local osteon arrangements [99,100,101,102]. Different osteon morphotypes, characterized by different local collagen orientations, have indeed been observed in different anatomical regions and their remodeling-driven fiber arrangement correlated to the local stress field [103].

**Figure 6:**
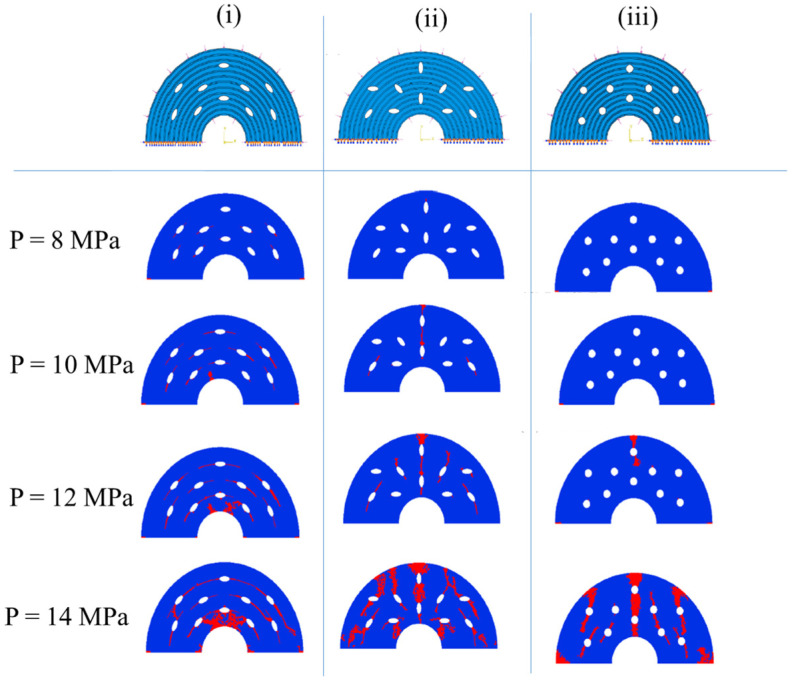
The influence of interstitial matrix and Haversian canal pressure on the crack behavior in osteons with different microdamaged conditions: (**i**) annularly elliptical lacunae structure; (**ii**) haphazardly elliptical lacunae structure; (**iii**) circular lacunae. XFEM damage status is represented in red. Reproduced with permission from [104]; copyright Elsevier.

As described until now, the most studied type of crack in an osteon is in the transversal direction where the path propagates toward or around the osteonic structure. Nevertheless, microporous damage, localized inside the osteonic region, can also cause complications. Knowing that these regions can have multiple configurations, Yin et al. [104] simulated three kinds of varying shapes (i.e., circular or elliptical) and different orientations (Figure 6). External conditions were set as constant pressure from the interstitial matrix and the Haversian canal. By changing the pressure from 8 MPa to 14 MPa, the study demonstrated that elliptic osteons can modify the crack path and minimize its impact on material failure. For example, it can make the crack propagate along the annular direction (Figure 6(i)) rather than toward the Haversian canal (Figure 6(iii)).

**Figure 7:**
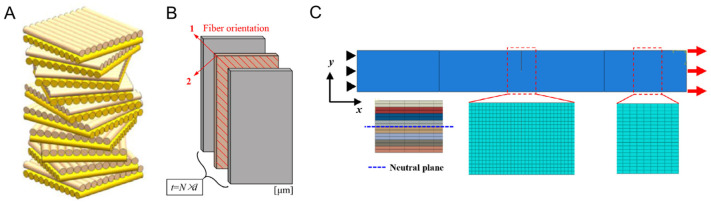
Double-twisted Bouligand structure. (**A**) Design schematic. (**B**) Fiber-oriented layer stacking: example of in-plane fiber orientation (1–2). (**C**) Simulation conditions: single-edge notched part under tensile stress. Reproduced with permission from [107]; copyright Elsevier.

Single-twisted Bouligand structures are described as helicoidal arrangements oriented across the thickness of the material. This arrangement can be observed in a variety of biological materials such as in the mantis shrimp dactyl club and the locust exoskeleton. This particular structure can create a nest of multiple twisting microcracks, locally releasing energy upon external stresses, and a macroscopic failure [105]. The scales of the coelacanth fish (*Latimeria chalumnae*) go beyond: their inner structures have overlapping helicoidal configurations, creating a double-twisted Bouligand structure (Figure 7A) and enhancing the fish defense capability [106]. Yang et al. [107] performed 3D XFEM simulations of Bouligand-like composites, where each stacked layer is assigned a different fiber orientation (Figure 7B). Not modeling individual-oriented fibers can be seen as a large simplification. However, it is an engaging approach to overcome the drawbacks of 3D XFEM simulations, where convergence issues are routinely present. Moreover, it facilitates finite element modeling. By studying orientations, commonly seen in single- and double-twisted Bouligand structures under tensile conditions (Figure 7C), the authors highlighted that the double configuration can achieve higher toughness than the single configuration by reducing intralaminar damage. Moreover, the pitch angle of the fibers (the angle variation between fibers of adjacent layers) directly influences the material behavior.

**Figure 8:**
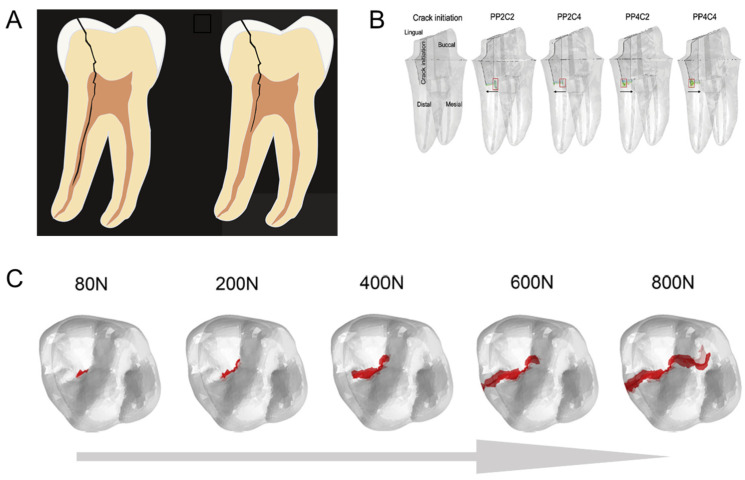
Crack propagation on teeth. (**A**) Deep and proximal radicular cracks, reproduced with permission from [112]; copyright John Wiley and Sons. (**B**) Different conditions can modify the crack propagation direction. Reproduced with permission from [113]; copyright Elsevier. (**C**) Crack path in a natural tooth with growing bite forces, reproduced with permission from [115]; copyright Elsevier.

The XFEM has also been demonstrated as a useful tool in applied dentistry. Perhaps the first publication in the field, Barani et al. [108] applied the XFEM to study the influence of axial forces acting during cracking of the tooth enamel. Despite being a pioneer, the authors still had to pre-set the crack location and size. Further, the same group revisited the method. First, they demonstrated that elongated teeth have higher resistance to longitudinal fracture [109]. Then, Barani et al. [110] evidenced that teeth with graded elastic moduli can increase crack segmentation. Finally, they simulated the splitting of molar teeth using the 3D XFEM, concluding that dentin toughness is more important than strength to fracture evolution [111]. Deep and proximal cracks (Figure 8A) in the radicular parts of the teeth can either originate from the crown or the root. Nevertheless, they require intervention to preserve the teeth and to avoid unnecessary extraction [112]. One preservation method is the usage of a composite resin core, with materials with a similar elastic modulus to the teeth dentin to minimize local stress concentration. Boonrawd et al. [113] used the XFEM to display the influence of the resin core on altering possible crack initiation and propagation direction (Figure 8B). Zhang et al. [114] explored the fracture resistance of a molar tooth with carious lesions and what is the best restoration method. With the aid of 3D XFEM simulations, the authors were able to compare conservative to more invasive restoration methods, highlighting that conservative methods involve less material removal, thus maintaining the tooth fracture resistance. In the same field, Zhang et al. [115] showed, through XFEM 3D modeling, how a crack propagates within a natural tooth upon growing bite forces (Figure 8C).

### 3.4. XFEM and Bioinspired Materials

**Figure 9:**
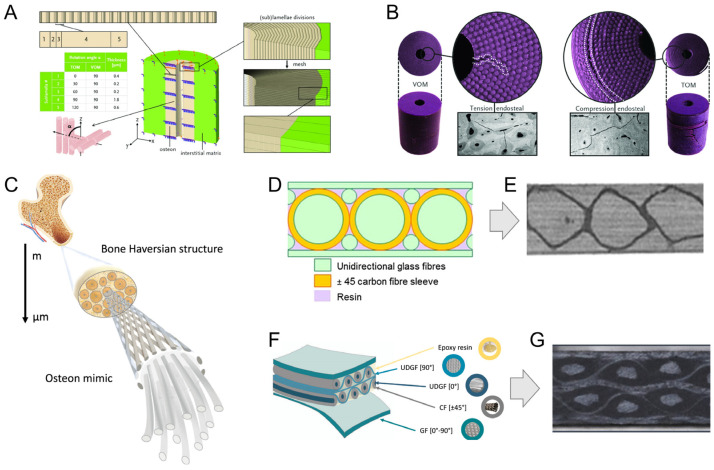
Inspiration and fabrication of bone-inspired composites. (**A**) The stacking sequence of vertical and twisted osteon morphotypes, VOM and TOM, respectively. (**B**) The failure modes of these composites, shown in 3D printed samples of VOM and TOM, and compared to the natural counterparts: i.e., Tension endosteal region and Compression endosteal region, respectively (grayscale microscopy pictures). (**A**,**B**) Reproduced with permission from [103]; copyright John Wiley and Sons. Cortical bone-inspired fiber-reinforced composites. (**C**) Inspiration from the Haversian structure, reproduced from [123] under CC BY 4.0 DEED. (**D**) First design and (**E**) fabricated composite (**D**,**E**). Reproduced with permission from [121]; copyright John Wiley and Sons. (**F**) Posterior design, with transversally orientated glass fibers reinforcing the bending characteristics. Reproduced from [123] under CC BY 4.0 DEED. (**G**) Cross-section of the final structure. Adapted from [123] under CC BY 4.0 DEED.

Humans are surrounded by biological materials that have established highly optimized functions after a billion-year-long evolution. Bioinspiration refers to extracting knowledge from nature-made systems, materials, or elements to create man-made ideas inspired by them. During the last few decades, encounters with bioinspired elements have been more present in daily life, from aerodynamical train designs based on the beak of a bird [116], to more efficient building ventilation inspired by termite mounds [117] and robot movements that mimic human demonstrations [118]. In the field of structural materials, where new bioinspired materials are daily developed, the material fracture behavior is essential to understanding its deformation and yielding mechanics [119,120]. In this sense, the XFEM is an important computational tool to predict the mechanical properties of bioinspired structures. Libonati et al. [103,121,122] showed that osteon morphotypes, thus variations in local collagen fiber organization, affect the overall material performance. The authors, using the FEM and 3D printing, highlighted that vertical morphotypes (VOM) perform better under tensile loading, and twisted morphotypes (TOM) withstand compression more effectively (Figure 9A,B) [103]. In further works, the authors showed the manufacturing of composite materials using carbon and glass fibers embedded into an epoxy matrix. The disposition and alignment of the fibers were positioned to resemble bone microarchitecture (Figure 9C). Specifically, the osteon and cement line were mimicked by unidirectional glass fibers enclosed by a carbon fiber sleeve (Figure 9D). Inspired by the interstitial lamellae, bundles of glass fibers were chosen to reinforce the space in between the osteons (Figure 9E) [121] and produced by applying peculiar fabrication methods. However, sub-par mechanical properties under bending stresses led to the adoption of transversally orientated glass fibers in a de novo design (Figure 9F), creating a potentially improved material (Figure 9G) [122].

**Figure 10:**
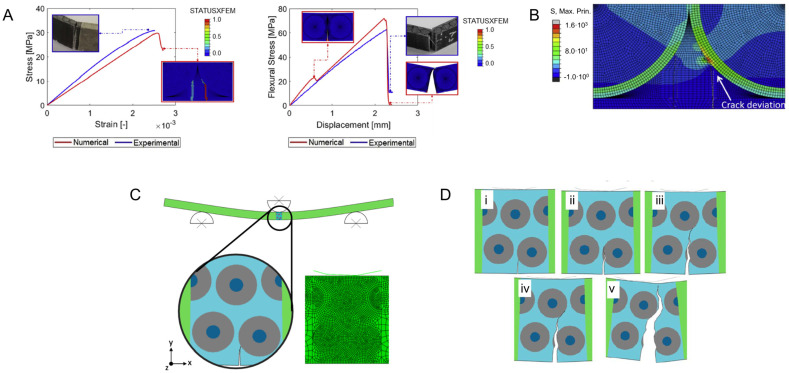
Bone-inspired composite analyzed through XFEM. (**A**) Mechanical behavior of the composite under tension and bending conditions, highlighting a good agreement between the XFEM (in red) and experimental (in blue) results. Reproduced with permission from [124]; copyright Elsevier. Inset depicting the experimental failure mode under three-point bending loading, reproduced with permission from [121]; copyright John Wiley and Sons. (**B**) The simulation shows the capacity of the osteonic structure to deviate and arrest the crack. Reproduced with permission from [124]; copyright Elsevier. (**C**) The de novo bioinspired design under bending conditions, showing (**D**) the crack propagation path during the bending test (**i**–**v**). (**C**,**D**) Reproduced from [123] under CC BY 4.0 DEED.

Vellwock et al. [124] and Libonati et al. [123] investigated these composite designs with the aid of the XFEM. As shown in Figure 10A, the computational model of the initial composite design demonstrated great agreement with the experimental results obtained through tensile and three-point bending tests. During the tensile test simulation, the initiated crack was deviated and arrested after contact with the cement-like structure (Figure 10A). In the three-point bending, the crack propagated in-between two osteonic regions, following an equal path seen during experimental tests (Figure 10A). Moreover, the XFEM reinforced the role of the osteon-like region in delocalizing stresses (Figure 10B). The second study focused on the fracture behavior of the composite with additional interlayered glass fibers under external bending stresses (Figure 10C), where the crack started in the expected region, on the lower surface of the composite. Then, it grew upwards, partially deviated by an osteon-inspired region. Following this, the crack was arrested and completely deflected by another osteonic structure. The XFEM analysis not only reproduced the experimental condition [123] but also highlighted fracture toughening mechanisms that can be translated to other materials designs.

Tang et al. [125] investigated a nacreous-like structure with the aid of the XFEM. Using brick-and-mortar structures with varying structural parameters, such as thickness, spacing, and aspect ratio, the study evidenced that the toughness and strength of a nacreous bioinspired material are mostly influenced by the microstructural aspect ratio. The model was validated based on experimental data from Barthelat et al. [126], showing a significantly low deviation from it.

**Figure 11:**
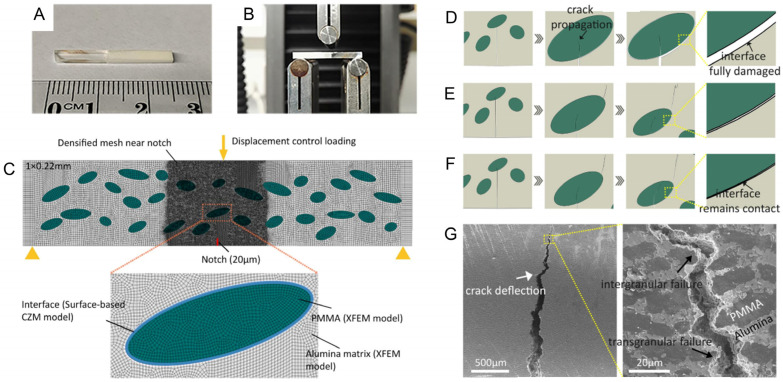
Freeze-casted composite made of PMMA and alumina. (**A**) Fabricated specimen. (**B**) Interfacial strength test setup. (**C**) XFEM+CZM model of the bioinspired composite. Computed fracture behavior under (**D**) low, (**E**) medium, and (**F**) high interfacial strength. (**G**) Experimentally observed failure mechanism. Reproduced with permission from [127]; copyright Elsevier.

Liu et al. [127] created freeze-cast composites inspired by graded architectures occurring in natural composites (Figure 11A). Applying a combination of the XFEM and the CZM, the material was studied under bending stresses (Figure 11B). The microarchitecture was composed of elliptical polymethyl methacrylate (PMMA) inclusions in an alumina matrix. Both have been modeled as XFEM-enriched regions, while their interfaces were set as surface-based CZM (Figure 11C). The results showed that the interface strength alters the fracture behavior significantly. At a low interfacial strength (i.e., 5 MPa) (Figure 11D), there is a fully interfacial delamination. Doubling the strength (i.e., 10 MPa) creates a partially damaged interface and, in addition to the main crack, a second crack initiation above the PMMA inclusion (Figure 11E). At an even higher interface strength (i.e., 15 MPa), interface damage is almost non-existent, and the main crack can propagate further into the PMMA region (Figure 11F). Nevertheless, the secondary crack path is similar to the model with medium interface strength. The models with medium and high strength are in great agreement with the fracture path observed in microscopy (Figure 11G), where crack deflection is the most observed fracture toughening mechanism.

**Figure 12:**
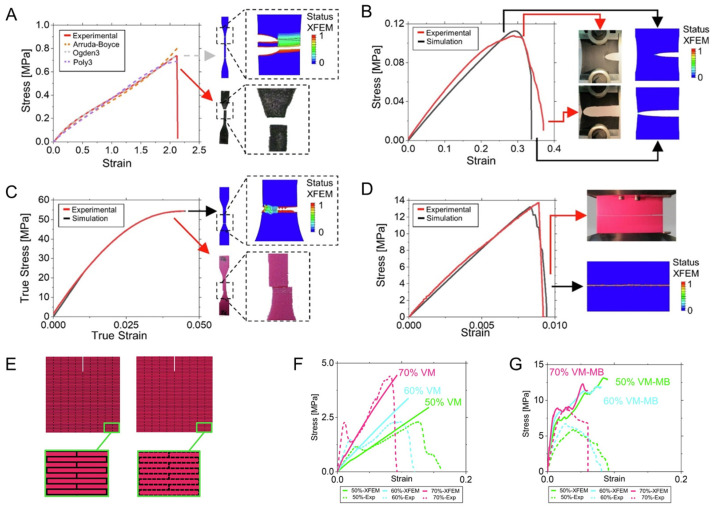
Fracture analyses on nacre-inspired material made with two components. (**A**–**D**) Single component results. Comparison between XFEM and experimental results for two testing modes: (**A**,**C**) uniaxial tensile and (**B**,**D**) fracture test. The research considered two single materials: (**A**,**B**) TangoBlackPlus, a soft hyperelastic material, and (**C**,**D**), VeroMagenta, a stiff linear elastic material. (**E**) Schematic of the nacre-inspired composite. (**F**,**G**) Mechanical behavior of the composites (**F**) without and (**G**) with mineral bridging. (**A**–**D**,**F**,**G**) Reproduced from [128] under Creative Commons CC-BY. (**E**) Adapted with permission from [129]; copyright Elsevier.

Recently, Aguilar Coello et al. [128] demonstrated the influence of mineral bridges in a nacre-inspired structure, where the matrix was modeled as a soft hyperelastic material and the filler as a stiff linear elastic material (Figure 12). Despite the usage of non-linear material properties, the authors highlighted the influence of the biomimetic mineral volumetric fraction on the overall fracture behavior, with the aid of the XFEM to simulate single (Figure 12A–D) and multi-materials (Figure 12E,F). However, the study evidenced that crack propagation was less problematic on single material specimens. The study was possibly the pioneer in applying the XFEM to simulate complex designs with hyperelastic components.

## 4. Future Outlook

This review introduced the XFEM formulation and its integration into commercial software and focused on discussing its application to composites, but mostly to biological and bioinspired materials. It is essential to emphasize that the XFEM has also been demonstrated in other fields such as in rockfill dams [130], concrete dams [131,132], bridges [133], and lithium-ion batteries [134,135]. In this review, the outlook section is structured point-wise, with perspectives for different research fields but also for the method itself.

- The XFEM and batteries. With the continuous growth in the batteries sector, it is expected that the usage of the XFEM in battery design will also increase, mainly predicting possible failure regions, resulting in a direct increase in battery life. For instance, Zhu et al. [134] applied the XFEM to show diffusion-induced fracture on lithium-ion batteries, highlighting that crack onset and growth is related to the current battery density.
- XFEM and fatigue load. Most of the studies discussed in this review have implemented static loads in their models. However, in the last few years, studies have started to combine cyclic loading and the XFEM [136,137,138,139]. For example, Gairola and Jayaganthan [139] presented a detailed study of the fatigue behavior of an aluminum alloy, employing the XFEM to predict the fatigue growth rate. With the expanded necessity to simulate structures under cyclic conditions (e.g., in orthopedic implants, and batteries), an upsurge in research and publications is likely in the next years.
- The XFEM and artificial intelligence (AI). In the medium term, the merging of AI techniques is envisioned. For instance, optimization techniques based on neural networks can use the outputs of XFEM simulations to improve structural designs, accelerate the design phase, and expand the boundaries of the design space. As reported in this review, small variations in the material properties of a composite component can widely affect the failure behavior. Nevertheless, these properties often have uncertainties associated with them, such as simplifications and difficulty in measuring exact values. In this direction, Bayesian networks can be helpful by introducing probability distributions of specific variables, such as material elastic modulus and yield strength, porosity distribution, or flaw orientation, thus creating a better understanding of the material mechanical behavior. XFEM models with Bayesian networks have been recently proposed [140], but generally lead to overly simplified structures. With the growth of the XFEM’s popularity, a combined dataset of published results can be used as input for data-driven models (i.e., meta-analysis). Meta-analyses are popular in other fields, especially in biological and medical research [141,142,143]. Yet, their utilization in applied science such as structural materials design is often neglected.
- The XFEM and convergence. Despite all of the positive results, current implementation of the XFEM into commercial software tends to yield convergence issues. These can be more pronounced when simulating crack initiation and propagation on intricate material architectures subjected to complex loads. To reduce their influence, a few inputs can be modified such as to check for deformed mesh elements (i.e., use adaptative meshing if large deformations are expected), decrease the minimum increment size of the time step, increase the maximum number of increments, allow for the accounting of geometric non-linearity (e.g., set automatic stabilization factors) and modify the general solution controls (i.e., increase the time incrementation factors). The inclusion of a pre-established crack is also open for debate. While it facilitates model convergence, it may lead to misleading results. For example, a pre-set crack in a region of the model where the crack initiation parameter would not be previously achieved could result in an incorrect overall failure behavior. These intrinsic computational limitations of the XFEM are its major drawback, not only in terms of the problematic with convergence, but as it works on a continuum scale, its embedding into a multiscale framework is challenging. Thus, possible positive effects from the inspiration on natural materials (e.g., material hierarchy) can be obscured.

## 5. Conclusions

The mere possibility of catastrophic failures of components and infrastructures is a substantial issue, drawing attention to the usage of safer designs and materials. Moreover, the growing interest in fracture assessment and fail-safe approaches (e.g., damage-tolerant designs) has put major attention on fracture analysis research. Nature applies smart microstructural design to increase material toughening mechanisms. Meanwhile, with the rise of biomimicry, engineering designs have taken it as inspiration to create better structural components using synthetic materials. Yet, predicting fracture behavior is a fundamental part of the biomimetic design of structural materials. The use of the XFEM has been highlighted as an established route to understanding how a material design can lead to a fail-safe approach, facilitating the creation of improved materials and the design of novel topological patterns in materials, especially composites. The method is a powerful tool for understanding the mechanical behavior in structural materials, from composites to biological and bioinspired materials. Its applications are growing considerably in many fields, but the method’s capacity is being retained due to computational issues. In the near future, further research is certainly needed to overcome these issues. Leveraging the increasing computer power and the continuous advancement in AI, new challenges are expected to be overcome.

## Figures and Tables

**Table 1:** Comparison of numerical methods that simulate fracture behavior.

Numerical Method	Pros	Cons
Contour Integral Method (CIM)	Robustness; Fast calculation speed; Simplicity.	Requires the modeling of the crack plane or line; Requires the specification of the crack front; Requires careful mesh preparation [7].
Virtual Crack Closing Technique (VCCT)	Available on commercial software.	Numerically challenging method, focused on the simulation of brittle fracturing [9].
Cohesive Zone Element (CZM)	Moderate numerical convergence [13].	Requires the pre-set of the crack propagation path [14].
Phase-Field Model (PFM)	Smooth transition from the intact to the damaged element; It does not consider the crack as a physical discontinuity; Possibility of multiple crack modeling and their branching and merging [19,20].	Diffuse damage profile due to regularization length [22,23]; Direct implementation into commercial software is absent [20,24,25].
eXtended Finite Element Method (XFEM)	It does not require the pre-set of crack parameters such as location, length, or propagation path [26,27,28,29].	Numerical convergence.

## Abbreviations

AI	Artificial Intelligence
CZM	Cohesive Zone Element
CIM	Contour Integral Method
FEM	Finite Elements Method
PFM	Phase-Field Model
PMMA	Polymethyl methacrylate
TOM	Twisted Morphotypes
VCCT	Virtual Crack Closing Technique
VOM	Vertical Morphotypes
XFEM	eXtended Finite Element Method
